# Interfacial Friction-Controlled Fiber Failure Modes for Toughness Enhancement of Engineered Cementitious Composites

**DOI:** 10.3390/ma19081643

**Published:** 2026-04-20

**Authors:** Dachuan Zhang, Yingzi Yang, Zhendi Wang, Ling Wang

**Affiliations:** 1 China Building Materials Academy, Beijing 100024, China; zhangdachuan0922@163.com (D.Z.); wangling@cbmamail.com.cn (L.W.); 2 School of Transportation Science and Engineering, Harbin Institute of Technology, Harbin 150090, China; 3 School of Civil Engineering, Harbin Institute of Technology, Harbin 150090, China; yzyang@hit.edu.cn

**Keywords:** Engineered Cementitious Composites (ECC), fiber–matrix interfacial frictional stress, fiber failure mode, micromechanical design, toughness

## Abstract

**Highlights:**

- Fiber–matrix interfacial frictional stress (*τ*_0_) governs the transition between fiber pull-out and fracture in ECC.
- A critical *τ*_0_ value is theoretically identified, at which fiber bridging achieves maximum energy dissipation.
- Toughness of ECC is maximized when *τ*_0_ approaches its critical value, as validated experimentally.

**Abstract:**

Despite extensive advancements in Engineered Cementitious Composites (ECCs), mixture design remains predominantly empirical, due to the absence of a quantitative parameter directly linking fiber–matrix interfacial mechanics to strain-hardening performance. This study identifies fiber–matrix interfacial friction as a quantifiable parameter and establishes a micromechanics-guided interfacial regulation framework to enhance the toughness of ECC by regulating fiber failure modes. First, a critical fiber–matrix interfacial frictional stress, (*τ*_0_)_crit_, corresponding to the transition between fiber pull-out and fracture, was theoretically derived based on energy dissipation maximization during crack propagation. A back-calculation approach was further developed to determine interfacial frictional stress (*τ*_0_) directly from tensile stress–crack opening responses under single-crack tension, eliminating reliance on single-fiber pull-out testing. Then, *τ*_0_ was tuned toward (*τ*_0_)_crit_ through interfacial regulation using fly ash. Experimental results demonstrate that the toughness of ECC is maximized when *τ*_0_ approaches (*τ*_0_)_crit_, confirming the validity of the proposed toughness enhancement mechanism. The study establishes an explicit mechanistic linkage between interfacial micromechanics and macroscopic strain-hardening performance, providing a predictive and quantitative design pathway that transcends empirical mixture adjustment.

## 1. Introduction

Engineered Cementitious Composites (ECCs), first proposed by Victor C. Li in the early 1990s, represent a family of ultra-ductile fiber-reinforced cementitious materials designed through a micromechanics and fracture mechanics-based framework [1]. The distinguishing feature of ECC is its metal-like tensile strain-hardening behavior, referred to as pseudo strain-hardening (PSH), accompanied by the formation of numerous tightly controlled microcracks less than 300 μm (maximum durability limit) [2]. The mix proportions and mechanical properties of the benchmark ECC, designated as “ECC-M45”, are shown in Table 1 and Table 2, respectively [3]. In contrast to conventional concrete, where tensile strain capacity generally remains below 0.02%, ECCs can reach ultimate tensile strains exceeding 3% [4,5], enabling applications requiring extremely high toughness.

The strain-hardening behavior of ECC originates from the synergy between fiber bridging capacity and controlled matrix cracking. For strain-hardening to occur, two prerequisites must be satisfied: the strength criterion for crack initiation and the energy criterion for steady-state crack propagation [6], as shown in Equations (1) and (2). These two criteria ensure that the composite develops multiple microcracks rather than a single catastrophic crack.

(1) $$\sigma_{fc}<\sigma_{0}$$

(2) $$J_{tip}<{J_{b}}^{\prime }$$

where $\sigma_{0}$—fiber bridging capacity, i.e., the ultimate tensile stress derives from single-crack uniaxial tensile test (MPa);

$\sigma_{fc}$—first crack strength, obtained by uniaxial tensile test (MPa);
$J_{tip}$—crack tip toughness (J/m);
${J_{b}}^{\prime }$—the maximum complimentary energy, calculated as the area to the left of *σ*-*δ* curve up to peak load (J/m^2^).

**Figure 1:**
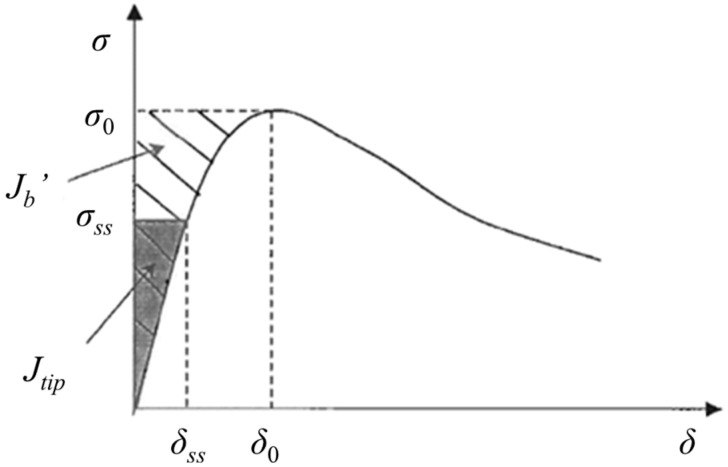
Typical *σ*-*δ* curve for ECC [7].

$J_{tip}$ and ${J_{b}}^{\prime }$ can be calculated using Equations (3) and (4) and depicted in the stress–crack opening (*σ*-*δ*) curve for a specific crack in an ECC, as illustrated in Figure 1.

(3) $$\sigma_{ss}\delta_{ss}-\int_{0}^{\delta_{ss}}\sigma (\delta )d\delta =J_{tip}\approx \frac{K_{m}^{2}}{E_{m}}$$

(4) $${J_{b}}^{\prime }=\sigma_{0}\delta_{0}-\int_{0}^{\delta_{0}}\sigma (\delta )d\delta$$

where *σ_ss_*—steady state bridging stress (MPa);

*δ_ss_*—steady state crack opening corresponding to *σ_ss_* (mm);
*σ*(*δ*)—*σ* as a function of *δ*;
${J_{b}}^{\prime }$—maximum complementary energy (J/m),
*K_m_*—fracture toughness of matrix (MPa ⋅ m^0.5^);
*E_m_*—elastic modulus of matrix (MPa).

To quantify the material’s ability to meet these criteria, PSH indices based on strength (*PSH_s_*) and energy (*PSH_e_*) have been widely adopted [8], as provided in Equations (5) and (6). In practice, PSH indices exceeding 1 provide the theoretical threshold for strain-hardening behavior, whereas substantially higher values are required to achieve saturated strain-hardening behavior, i.e., measured/theoretical value of average crack spacing ≤ 2. For example, indices for PVA-based ECC, should meet *PSH_s_* > 1.3 [9] (1.45 [10]) and *PSH_e_* > 2.7 [7]. For PE-based ECC, *PSH_s_* > 1.2 [11,12] and *PSH_e_* > 3 [11] should be satisfied. Higher PSH indices indicate greater potential for exhibiting strain-hardening behavior, which is typically associated with increased ultimate tensile strain [13,14].

(5) $$PSH_{s}=\sigma_{0}/\sigma_{fc}$$

(6) $$PSH_{e}={J_{b}}^{\prime }/J_{tip}$$

With crack opening increase, fibers in the ECC first debond and then slip off the matrix. Thus, the fiber–matrix interfacial friction governs both the strength and energy requirements of strain-hardening behavior by determining the fiber failure mode, directly influencing the toughness of ECC. Excessively strong interfaces promote fiber rupture, leading to brittleness and suppressed strain-hardening behavior. Conversely, overly weak interfaces limit fiber strength mobilization, lowering fiber bridging capacity ($\sigma_{0}$) and toughness. Achieving a well-balanced interfacial friction is therefore fundamental to enhancing toughness.

Substantial research has sought to tune interfacial properties through fiber or matrix modifications. Hydrophobic fibers such as polyethylene (PE) and polyethylene terephthalate (PET) fiber are typically surface-functionalized with materials including graphene oxide (GO) [15], acrylic resins [16], carbon nanofibers (CNFs) [17], or polydopamine (PDA) [18,19] to increase interfacial friction and improve load transfer. Hydrophilic polyvinyl alcohol (PVA) fibers, on the other hand, often require oil-based surface treatments to intentionally reduce interfacial friction [20] and suppress undesirable fiber rupture.

In comparison, modifying the matrix provides a more convenient and economical approach to tailoring interfacial friction. The incorporation of supplementary cementitious materials (fly ash [21,22], slag [23], limestone powder [24], calcined clay [25], etc.) and solid waste materials (glass powder [26], rubber particles [27,28], etc.) has been shown to modify matrix reactivity, porosity and microstructure, thereby affecting fiber–matrix interfacial friction.

Conventional ECC design, although based on micromechanical principles and PSH criteria, primarily regulates the interfacial frictional stress (τ_0_) in a qualitative manner through empirical adjustment. No quantitative interfacial descriptor has been established to directly guide the mixture design of ECCs. Consequently, interfacial regulation remains largely phenomenological rather than predictive.

To address this gap, this study explicitly treats τ_0_ as a quantitatively controllable design parameter from a micromechanical perspective, aiming to establish a direct linkage between interfacial properties and macroscopic tensile performance. A back-calculation approach based on single-crack tensile response is developed to determine τ_0_, providing a practical alternative to conventional single-fiber pull-out tests. It is further demonstrated that an optimal τ_0_ exists at the transition between fiber rupture and pull-out, at which the tensile toughness of ECC is maximized. An analytical formulation is derived to determine this value, thereby establishing τ_0_ as a mechanism-governing and quantitatively designable parameter. Finally, fly ash is employed to systematically regulate interfacial friction, and the relationship among τ_0_, fiber failure mode, and toughness is experimentally validated.

## 2. Mix Design, Materials, and Experimental Program

### 2.1. Toughness Enhancement Mechanism

Under tensile loading, cracking initiates within the ECC matrix. As the crack opening displacement (*δ*) increases, fibers bridging the crack progressively carry increasing tensile stress, leading to interfacial debonding between the fiber and matrix. The debonded portion of the fiber subsequently begins to slip. As the debonded length increases, the interfacial frictional resistance increases due to the enlargement of the contact area between the fiber and matrix.

Depending on the interfacial friction level, two distinct fiber failure modes may occur during crack bridging: fiber fracture and fiber pull-out. Fiber fracture occurs when the tensile stress in the fiber exceeds its tensile strength during interfacial debonding, whereas fiber pull-out takes place when the fiber fully slides out of the matrix without rupture.

For an individual fiber, a critical condition exists at the boundary between these two failure modes. Under this condition, the tensile stress in the fiber reaches its tensile strength exactly at the completion of interfacial debonding. At this point, the fiber simultaneously achieves full tensile capacity and maximum sliding displacement, resulting in maximum energy dissipation during crack propagation.

In ECCs, however, crack bridging involves a large population of fibers whose failure modes are governed by multiple factors, including fiber embedded length, inclination angle and fiber–matrix interfacial frictional stress (*τ*_0_) [29]. When fibers are uniformly distributed, *τ*_0_ plays a dominant role in controlling stress transfer. When *τ*_0_ is relatively low, fiber pull-out dominates; when *τ*_0_ is sufficiently high, fiber rupture becomes increasingly prevalent. The interfacial frictional stress therefore acts as a bifurcation parameter governing the transition between these two failure modes.

Accordingly, a critical interfacial frictional stress, denoted as (*τ*_0_)_crit_, can be defined. When *τ*_0_ ≤ (*τ*_0_)_crit_, all fibers bridging the crack preferentially undergo pull-out, whereas *τ*_0_ > (*τ*_0_)_crit_ inevitably leads to partial fiber fracture.

Greater toughness is achieved when *τ*_0_ approaches (*τ*_0_)_crit_. When *τ*_0_ is lower than this value, the fiber bridging stress is insufficient to fully mobilize the tensile capacity of the fibers. Conversely, when *τ*_0_ exceeds the critical value, premature fiber rupture reduces sliding energy and increases the brittleness of the composite. Therefore, maximizing the toughness of ECCs requires regulating *τ*_0_ toward the critical value (*τ*_0_)_crit_, thereby balancing ECC ductility and fiber strength mobilization.

### 2.2. Constituent Materials and Their Methodological Roles

**Figure 2:**
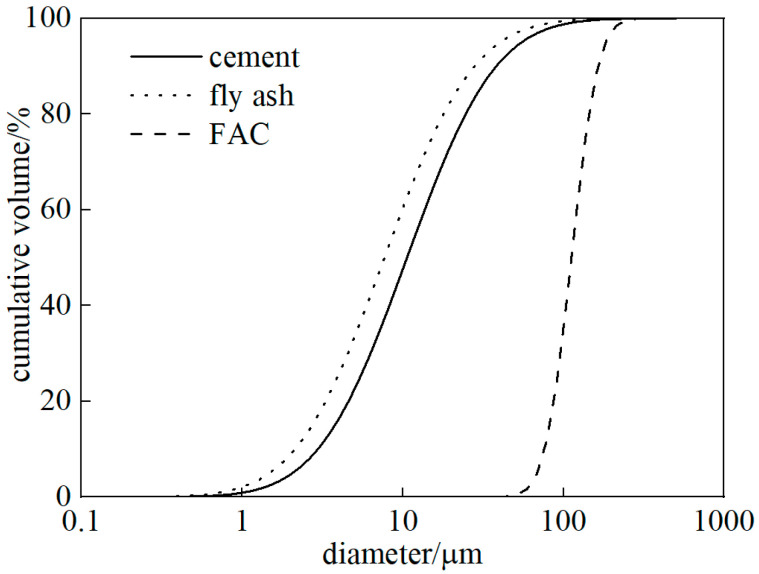
Particle size distribution of cement, fly ash and fly ash cenosphere.

CEM I 52.5 N cement, ASTM Class F fly ash and silica fume (Elkem Microsilica, Oslo, Norway) were employed as binder components. In this study, fly ash served as the primary variable to systematically tailor the fiber–matrix interfacial friction. Silica fume was incorporated to preferentially consume calcium hydroxide (Ca(OH)_2_) during early hydration, which rendered the regulatory effect of fly ash more efficient. Fly ash cenosphere (FAC) with an apparent density of 660 kg/m^3^ was regarded as an inert lightweight filler due to its limited pozzolanic reactivity [30]. FAC was introduced to generate distributed collective defects within the cementitious matrix, thereby reducing the matrix fracture toughness and facilitating steady-state crack propagation. The oxide compositions of cement, fly ash and FAC are summarized in Table 3 while their particle size distributions are shown in Figure 2. Polyvinyl alcohol (PVA) fibers (Kuraray K II, Osaka, Japan) were used as reinforcement, with an average diameter of 40 μm, a tensile strength of 1560 MPa, an elongation of 6.5% and an elastic modulus of 41 GPa. A polycarboxylate-based water-reducing admixture (WRA) and tap water were also incorporated into the mixtures.

### 2.3. Mix Proportions and Specimen Preparation

**Figure 3:**
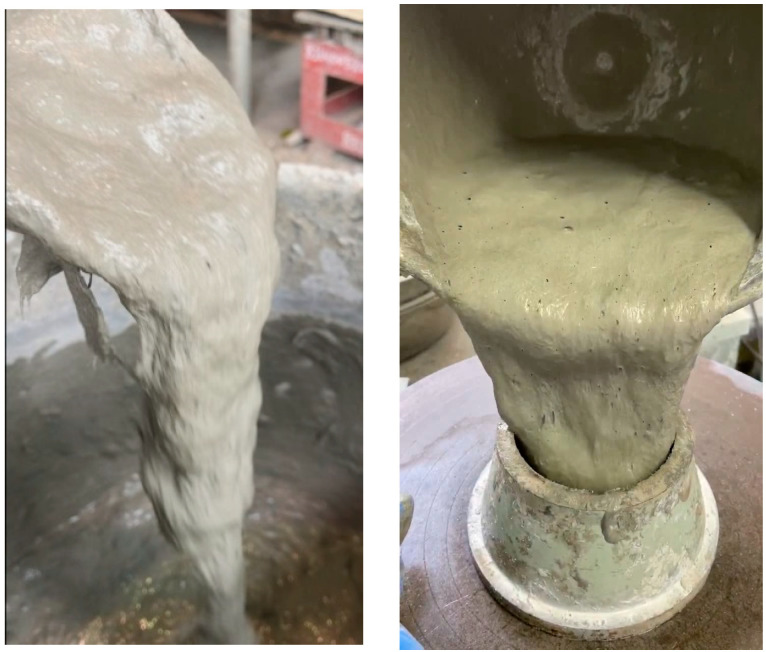
Flowability of ECC.

The mix proportions and designations of ECC mixtures are presented in Table 4. The numerical suffix of each mixture denotes the fly ash content, defined as the mass fraction of fly ash in the total binder. Five fly ash levels (0–50%) were selected to provide a continuous and sufficiently wide range for modifying interfacial characteristics. The water-to-binder ratio was fixed at 0.25, and silica fume was maintained at 5% of the total binder content. The filler-to-binder ratio was kept constant at 0.30. The fiber volume fraction was fixed at 1.2%, and the mass dosage of fibers was adjusted according to the measured density of each mixture to ensure accurate volumetric consistency. Workability was controlled by adjusting the dosage of WRA to achieve a target flow diameter of 200 ± 10 mm, measured in accordance with BS EN 1015-3 [31], as illustrated in Figure 3. This level of flowability ensured uniform fiber dispersion and allowed casting with only manual vibration.

All mixtures were prepared using a 5 L planetary mixer (Wuxi Jianyi Instrument & Machinery Co., Ltd., Wuxi, China). Dry powders were first blended at 140 r/min for 2 min to ensure homogeneity. A total of 50% of mixing water was then added, followed by 2 min of mixing. The remaining water containing pre-dissolved WRA was introduced and mixed for an additional 2 min, followed by 1 min at an increased speed of 285 r/min. PVA fibers were gradually added while mixing at 140 r/min for approximately 5 min to promote uniform fiber dispersion and avoid fiber agglomeration.

Fresh mixtures were cast into molds, compacted by manual vibration, and covered with plastic sheets. After 48 h, specimens were demolded and cured in a fog room at 20 ± 2 °C and relative humidity exceeding 90% until testing.

### 2.4. Test Methods and Procedures

#### 2.4.1. Single-Crack Tensile Test

**Figure 4:**
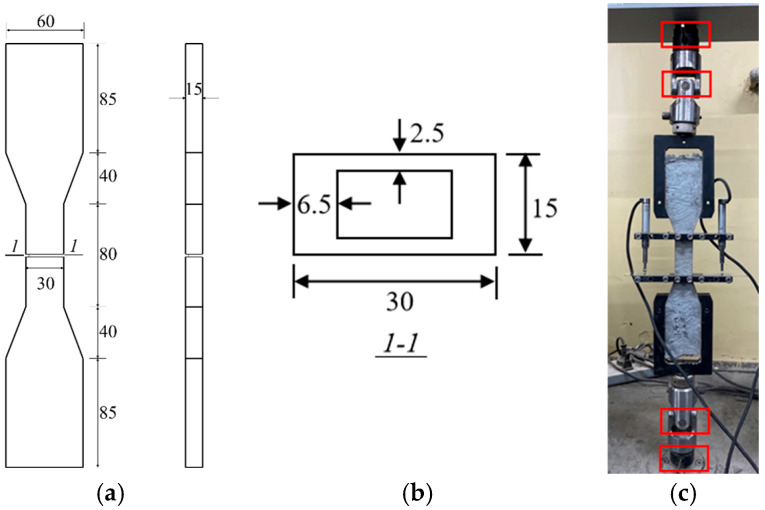
Set up and dimension of specimen of single-crack tensile test (mm). (**a**) Dimension of specimen; (**b**) Dimension of notch; (**c**) Set up.

Single-crack tensile tests were conducted at the 28th day to directly characterize the fiber bridging behavior and to quantify the fiber–matrix interfacial frictional stress. For each mix, 3 specimens were tested. The specimen geometry and test setup are illustrated in Figure 4. A pre-formed notch was introduced at the specimen mid-length of the specimen to localize crack initiation and enforce the development of a single dominant crack, thereby eliminating variability associated with multiple cracking patterns. To ensure true uniaxial loading conditions, two pivot joints were integrated into the loading fixtures, allowing automatic alignment of the applied load with the specimen axis. This configuration significantly reduces bending effects compared with conventional gripping systems. Axial deformation was measured using two linear variable displacement transducers (LVDTs) (Jinan Hengruijin Testing Machine Co., Ltd., Jinan, China) mounted on opposite sides of the specimen. Loading was applied under displacement control at a rate of 0.005 mm/s to simulate quasi-static conditions, consistent with previous studies [32]. The resulting stress–crack opening (*σ*-*δ*) relationships were used to characterize fiber bridging behavior and enable the back-calculation of the fiber–matrix interfacial frictional stress.

#### 2.4.2. Matrix Fracture Toughness Test

**Figure 5:**
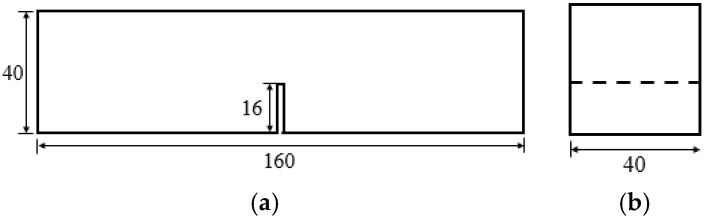
Dimension of specimen of matrix fracture toughness test (mm). (**a**) Main view; (**b**) Left view.

**Figure 6:**
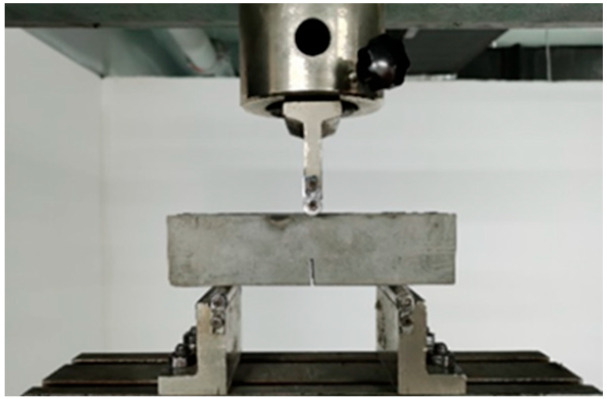
Device for matrix fracture toughness test.

The fracture toughness of the matrix was evaluated by a three-point bending test to obtain the matrix fracture parameters required for micromechanical analysis. For each mix, 3 specimens were tested. Prismatic specimens with dimensions of 40 mm × 40 mm × 160 mm were prepared with a centrally located notch of 16 mm depth. The specimen geometry and test configuration are shown in Figure 5 and Figure 6, respectively. The span length was set to 100 mm, and loading was applied at a displacement rate of 0.05 mm/min. The fracture toughness was calculated based on the maximum applied load using Equation (7).

(7) $$K_{m}=\frac{1.5(F_{Q}+\frac{mg}{2}\times 10^{-2})\times 10^{-3}\cdot S\cdot a_{0}^{1/2}}{th^{2}}f(\alpha )$$

where *F_Q_*—maximum load (kN);

*m*—mass of specimen (kg);
  *g*—acceleration of gravity, 9.8 m/s^2^;
  *S*—span of specimen (m);
*a*_0_—depth of notch (m);
*t*—thickness of specimen (m);
*h*—width of specimen (m);
*f*(*α*)—shape parameter of specimen,
  $$f(\alpha )=\frac{1.99-\alpha (1-\alpha )(2.15-3.93\alpha +2.7\alpha^{2})}{(1+2\alpha )(1-\alpha {)}^{1.5}},\;a=\frac{a_{0}}{h}.$$

#### 2.4.3. Thermal Gravity Test

Thermogravimetric analysis (TGA) was conducted using a PT1600 thermogravimetric analyzer (Linseis, Selb, Germany). For each mix, 1 specimen was tested. The samples were heated from room temperature to 1000 °C at a rate of 10 °C/min under a helium atmosphere, followed by a dwelling period of 15 min. The specimens were initially sealed at room temperature for 48 h and subsequently cured under standard conditions until 28 d. At 28 d, the specimens were crushed and ground into powder. Hydration was subsequently terminated by solvent exchange using absolute ethyl alcohol to replace the pore water completely. The samples were then dried in a vacuum desiccator at 40 °C until test.

## 3. Illustration of Micromechanical-Based Optimization Framework

### 3.1. Determination of the Critical Interfacial Frictional Stress (τ_0_)_crit_

To implement the proposed optimization strategy, it is essential to determine the critical interfacial frictional stress, (*τ*_0_)_crit_, corresponding to the transition between fiber pull-out and fracture.

For a single fiber bridging a crack, the tensile stress in the fiber increases progressively during interfacial debonding as the crack opening displacement enlarges. When the embedded length exceeds a critical value, the fiber ruptures before complete debonding. This critical embedded length, denoted as *l_u_*, can be derived from the equilibrium between fiber tensile strength and interfacial frictional resistance. Based on the micromechanical model proposed by Li et al. [1], *l_u_* can be expressed as follows [1]:

(8) $$l_{u}=L_{c}e^{-f\Phi }$$

(9) $$L_{c}=\frac{\sigma_{fu}d_{f}}{4\tau_{0}}$$

where *Φ*—fiber inclination (rad), the angle of fiber normal to failure plane;

*L_c_*—the critical embedded length of bridging fiber when Φ = 0 (mm);
*f*—snubbing coefficient, 0.2 [33];
  *σ_fu_*—effective tensile strength of fiber (MPa);
*d_f_*—diameter of fiber (mm);
*τ*_0_—frictional stress at fiber–matrix interface (MPa).

During fiber sliding, surface abrasion induced by matrix asperities may reduce the effective tensile strength of the fiber by approximately 10% relative to the nominal value provided by the manufacturer [34]. Accordingly, the effective fiber tensile strength *σ_fu_* adopted in this study was taken as 1404 MPa. Since the fiber inclination angle *Φ* varies between 0 and *π*/2, the corresponding *l_u_* ranges from *L_c_e*^−*fπ*/2^ to *L_c_*. To ensure that all fibers bridging a crack undergo pull-out rather than fracture, the minimum value of *l_u_* must be no less than the maximum possible embedded fiber length, as expressed by the following:

(10) $$\frac{\sigma_{fu}d_{f}}{4\tau_{0}}e^{-f\pi /2}\geq \frac{L_{f}}{2}$$

where *L_f_*—the length of fiber (mm).

It can be known from inequality (10) that $\tau_{0}\leq \frac{\sigma_{fu}d_{f}e^{-f\pi /2}}{2L_{f}}$. Substituting the material parameters into the above relationships yields the critical interfacial frictional stress ${\left(\tau_{0}\right)}_{crit}=\frac{\sigma_{fu}d_{f}e^{-f\pi /2}}{2L_{f}}=1.71$ (MPa).

### 3.2. Back-Calculation of Interfacial Frictional Stress τ_0_

The interfacial frictional stress (*τ*_0_) is conventionally determined using single-fiber pull-out tests [35]. However, such tests typically require fiber lengths exceeding 120 mm, whereas commercially available PVA fibers used in ECCs are only 8–12 mm in length. Therefore, direct measurement of *τ*_0_ via pull-out tests is often impractical.

To address this limitation, a micromechanics-based back-calculation approach was developed in the present study to estimate *τ*_0_ from single-crack tensile tests.

It should be noted that fiber orientation may influence the bridging behavior and thus the evaluation of τ_0_. In the present study, fibers are assumed to be randomly distributed with no preferential alignment, as the specimens were prepared using a conventional casting process. In addition, all mixtures exhibited good and consistent workability, as indicated by a flow diameter of 200 ± 10 mm, and the fresh ECC appearance (Figure 3) further confirms the uniform dispersion of fibers throughout the matrix.

According to the theoretical fiber bridging model proposed by Li et al. [1], the relationship between fiber bridging stress *σ* and crack opening displacement *δ* can be described by Equations (11)–(15). Neglecting slip-hardening effects, the ascending branch of the *σ*-*δ* curve, applicable to both fiber fractured and pulled-out, is governed by these equations, which relate *σ* and *δ* to interfacial and fiber parameters:

(11) $$\sigma =V_{f}\tau_{0}L_{f}/(2d_{f})\cdot g[2{\left(\frac{\hat{\delta }}{{\hat{\delta }}^{*}}\right)}^{1/2}-\frac{\hat{\delta }}{{\hat{\delta }}^{*}}]$$

(12) $$g=\frac{2}{4+f^{2}}(1+e^{\pi f/2})$$

(13) $$\hat{\delta }=\frac{2\delta }{L_{f}}$$

(14) $${\hat{\delta }}^{*}=2(\tau_{0}/E_{f})(L_{f}/d_{f})/(1+\eta )$$

(15) $$\eta =\frac{E_{f}V_{f}}{E_{m}(1-V_{f})}$$

where $V_{f}$—volume friction of fiber (%);

$E_{f}$—elastic modulus of fiber (MPa).

According to Equations (11)–(15), the bridging stress *σ* can be expressed as a function of *δ*, as shown in Equation (16), indicating that *σ* follows a quadratic relationship with *δ*^0.5^:

(16) $$\sigma =-\frac{(1+\eta )gV_{f}E_{f}}{2L_{f}}\delta +V_{f}g\sqrt{\frac{\tau_{0}(1+\eta )E_{f}}{d_{f}}}\sqrt{\delta }$$

Accordingly, *τ*_0_ can be determined through the following procedure:

(1) The *σ*-*δ* curve obtained from the single-crack tensile test is transformed into a *σ*-*δ*^0.5^ relationship;
(2) The transformed curve is fitted using a quadratic function, *y* = *Ax*^2^ + *Bx* + *C* where $A=-(1+\eta )gV_{f}E_{f}/(2L_{f})$, until convergence is achieved;
(3) Interfacial frictional stress *τ*_0_ is calculated from the fitted coefficient *B* according to Equation (17).

Because the tensile response prior to crack initiation is dominated by matrix elasticity rather than fiber bridging, the fitted curve typically deviates from the experimental data near the origin. Therefore, the constant term *C* is not constrained to zero [6].

(17) $$\tau_{0}=\frac{B^{2}d_{f}}{(1+\eta )E_{f}V_{f}^{2}g^{2}}$$

This procedure enables an indirect yet robust estimation of *τ*_0_ from macroscopic tensile measurements.

### 3.3. Modification of Interfacial Frictional Stress τ_0_

The fiber–matrix interfacial frictional stress *τ*_0_ was tailored by varying the fly ash content, with the objective of regulating *τ*_0_ toward the critical value (*τ*_0_)_crit_.

## 4. Results and Discussion

### 4.1. Fiber–Matrix Interfacial Frictional Stress: Regulation of τ_0_ Toward (τ_0_)_crit_

**Figure 7:**
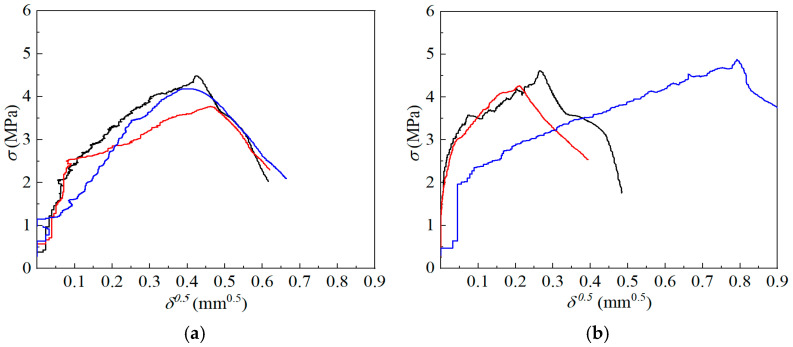

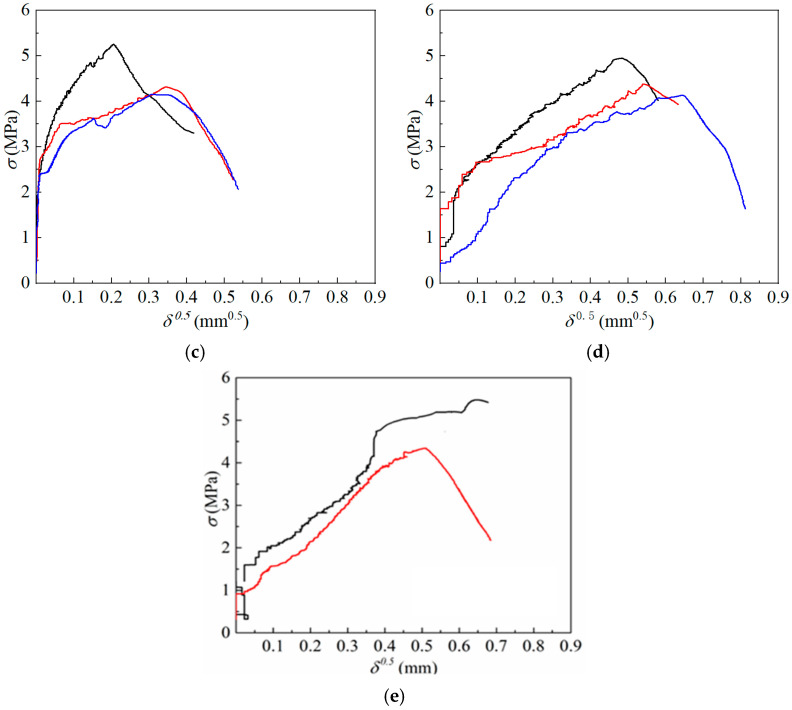
*σ*-*δ*^0.5^ curves. (**a**) FA0; (**b**) FA12.5; (**c**) FA25; (**d**) FA37.5; (**e**) FA50.

**Figure 8:**
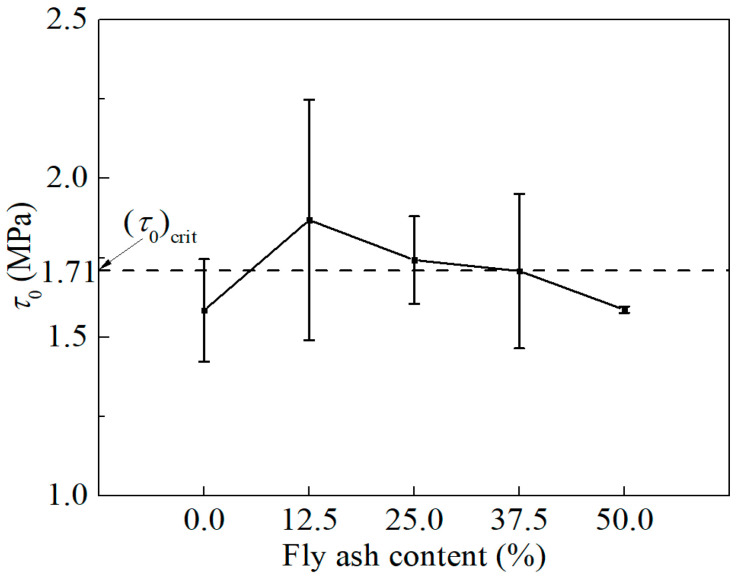
Frictional stress at fiber–matrix interface.

The tensile stress–crack opening displacement (*σ*-*δ*) responses of ECC mixtures with varying fly ash contents are presented in Figure 7, illustrating the influence of fly ash dosage on fiber bridging behavior. The corresponding *τ*_0_ values back-calculated from these curves are summarized in Figure 8. The calculated τ_0_ values show good agreement with those obtained from single-fiber pull-out tests [33]. In Ref. [33], where the same type of fiber was used and the fly ash content was 54.5%, τ_0_ was reported to range from 1.58 to 1.87 MPa for fiber volume fractions between 0.5% and 2%. In the present study, with a fiber volume fraction of 1.2% and a comparable fly ash content of 50%, τ_0_ is determined to be 1.59 MPa, which lies within the reported range. This consistency supports the validity of the proposed back-calculation approach.

For PVA-ECC, the optimal *τ*_0_ range is generally reported to be 1.0–2.0 MPa [10]. As shown in Figure 8, all mixtures fall within this range, indicating favorable conditions for strain-hardening performance. The incorporation of fly ash effectively modifies *τ*_0_, demonstrating that interfacial properties can be tuned through the regulation of fly ash content. For ECC0 (without fly ash), *τ*_0_ is significantly lower than the critical value (*τ*_0_)_crit_. With increasing fly ash content, *τ*_0_ exhibits a non-monotonic trend, initially increasing and subsequently decreasing. Notably, *τ*_0_ approaches (*τ*_0_)_crit_ most closely at a fly ash content of 37.5%, indicating the most favorable condition for enhancing toughness.

**Figure 9:**
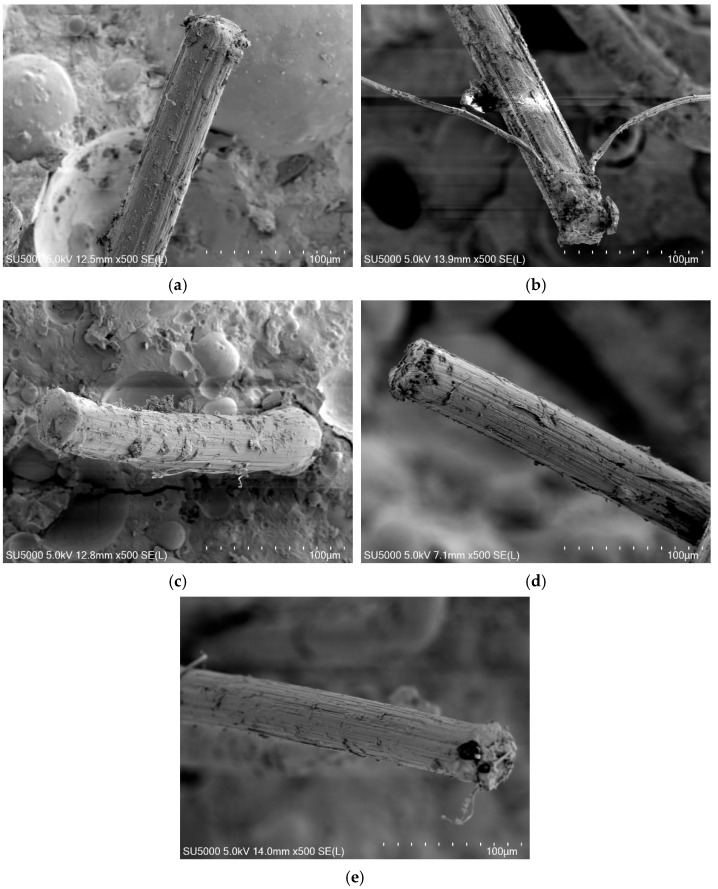
SEM images of fibers at fractured surface. (**a**) FA0; (**b**) FA12.5; (**c**) FA25; (**d**) FA37.5; (**e**) FA50.

SEM observations of fiber surfaces after pull-out (Figure 9) reveal varying degrees of surface abrasion, which qualitatively reflect differences in interfacial friction. More pronounced surface wear corresponds to higher interfacial resistance during fiber sliding, providing microstructural evidence supporting the evolution of *τ*_0_.

### 4.2. Uniaxial Tensile Performance: Validation of the Toughness Enhancement Strategy

The uniaxial tensile performance of each ECC mixture was systematically evaluated to verify the effectiveness of the proposed micromechanics-based interfacial optimization strategy. The results of the matrix fracture toughness tests are summarized in Table 5. Due to the similar densities of fly ash and cement, specimen mass remained nearly constant across mixtures; thus, variations in fracture toughness are primarily governed by peak load, as indicated by Equation (12). With increasing fly ash content, a clear monotonic decrease in fracture toughness is observed, which can be attributed to the dilution effect and the presence of unreacted fly ash particles that suppress early hydration products and weaken tensile resistance of the matrix [36].

The fiber bridging parameters obtained via single-crack tensile tests are presented in Table 6. The toughness is presented as fiber bridging energy absorption, quantified by $\int_{0}^{\delta_{0}}\sigma (\delta )d\delta$, exhibits a non-monotonic trend, increasing initially and then decreasing at higher fly ash dosages. The maximum value occurs at a fly ash content of 37.5%, indicating an optimal balance between bridging strength (*σ*_0_) and crack opening capacity (*δ*_0_). This observation directly validates the toughness enhancement mechanism proposed in Section 3.1, confirming that the energy dissipation capacity of ECC is maximized when *τ*_0_ approaches (*τ*_0_)_crit_.

**Figure 10:**
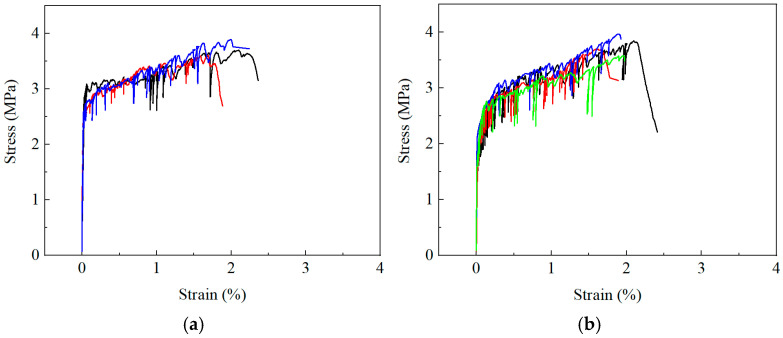

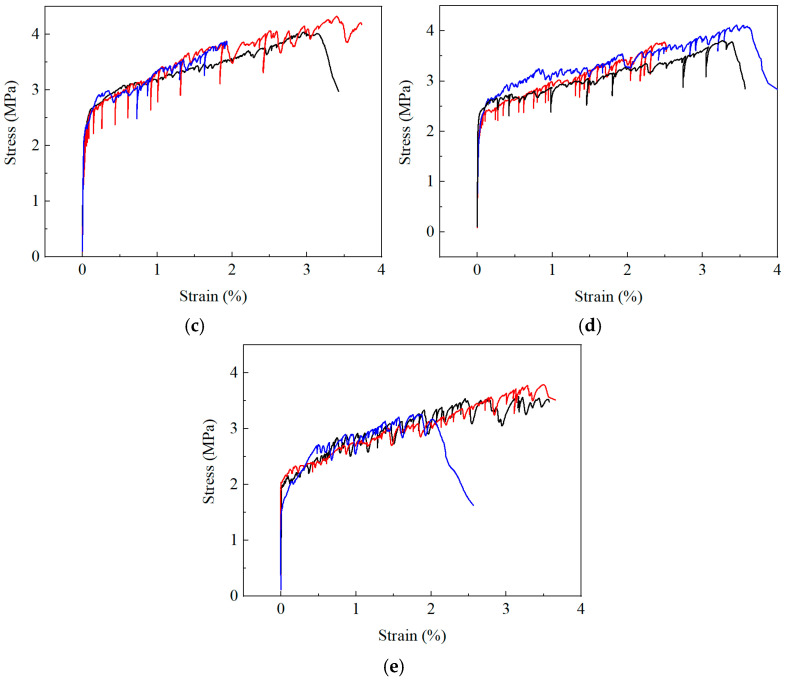
Tensile stress–strain curves [36]. (**a**) FA0; (**b**) FA12.5; (**c**) FA25; (**d**) FA37.5; (**e**) FA50.

The uniaxial tensile responses are summarized in Figure 10 and Table 7 [36]. The first cracking strength decreased with increasing fly ash content, consistent with the reduction in matrix fracture toughness. The ultimate tensile strain exhibits a pronounced non-monotonic evolution, peaking at intermediate fly ash content. The ultimate tensile strength follows a trend similar to that of *σ*_0_. It is worth noting that the ultimate tensile strength corresponds to the weakest crack plane in the specimen, whereas *σ*_0_ represents the bridging capacity at a controlled crack. Due to inevitable spatial heterogeneity in fiber distribution and lightweight inclusions, the ultimate tensile strength is consistently lower than *σ*_0_.

**Figure 11:**
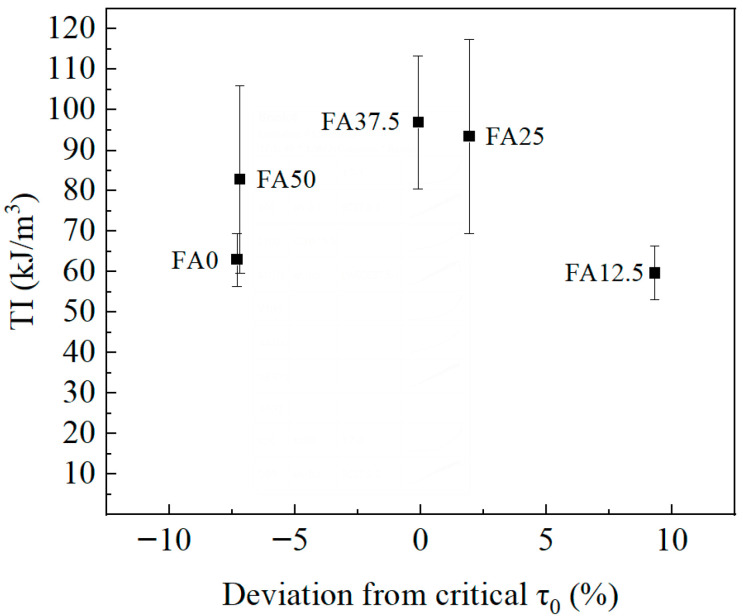
Relationship between normalized interfacial frictional stress deviation and toughness index.

The toughness index (TI) of ECC, defined as energy absorption capacity per union volume, was calculated from the tensile stress–strain curve using Equation (18). The relationship between the normalized deviation of interfacial frictional stress, expressed as (*τ*_0_ − (*τ*_0_)_crit_)/(*τ*_0_)_crit_ and TI, is presented in Figure 11. A smaller deviation corresponds to a higher TI value, further validating the effectiveness of regulating *τ*_0_ toward its critical value.

(18) $$TI=\frac{\int_{0}^{\varepsilon_{u}}\sigma (\varepsilon )d\varepsilon }{bdl}$$

where $\varepsilon_{u}$—strain corresponding to ultimate tensile stress;

b—width of tested zone (30 mm);
d—depth of tested zone (15 mm);
l—length of tested zone (80 mm).

Based on experimentally determined parameters characterizing matrix fracture and fiber bridging, the PSH strength index (*PSH_s_*) and energy index (*PSH_e_*) were calculated and are summarized in Table 8 and Table 9, respectively. Both indices increased significantly with the fly ash content, indicating enhanced propensity for stable multiple cracking and strain-hardening. All mixtures satisfy the theoretical criteria for saturated strain-hardening behavior (*PSH_s_* > 1.45 and *PSH_e_* > 2.7) [7,9,10].

### 4.3. Thermogravimetric Analysis: Hydration-Controlled Mechanism of τ_0_ Evolution

The interfacial frictional stress *τ*_0_ is positively correlated with the matrix compactness of ECC. Thermogravimetric analysis (TGA) was therefore conducted to quantify the contents of key hydration products, including calcium silicate hydrate (C-S-H), calcium hydroxide (CH), and ettringite (AFt).

C-S-H and AFt exhibit dense microstructures and high microhardness, contributing significantly to interfacial friction. In contrast, CH possesses a relatively loose crystalline structure with negligible mechanical contribution. Therefore, higher contents of C-S-H and AFt correspond to increased τ_0_.

**Figure 12:**
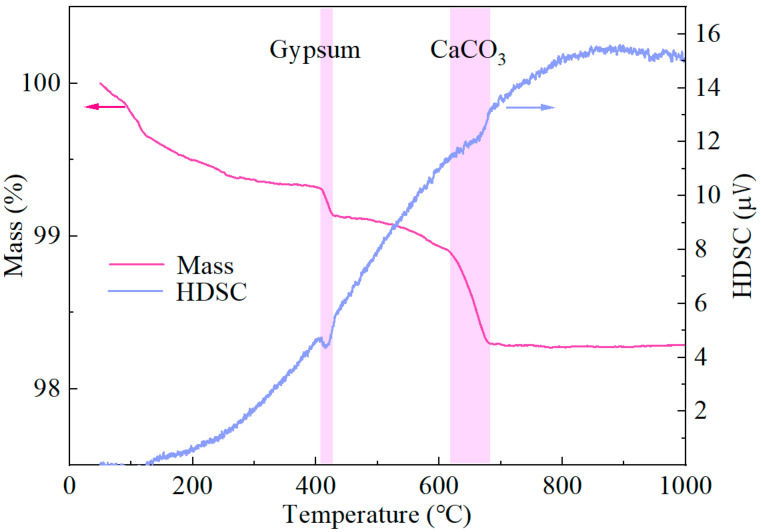
TG and HDSC curves of cement [36].

**Figure 13:**
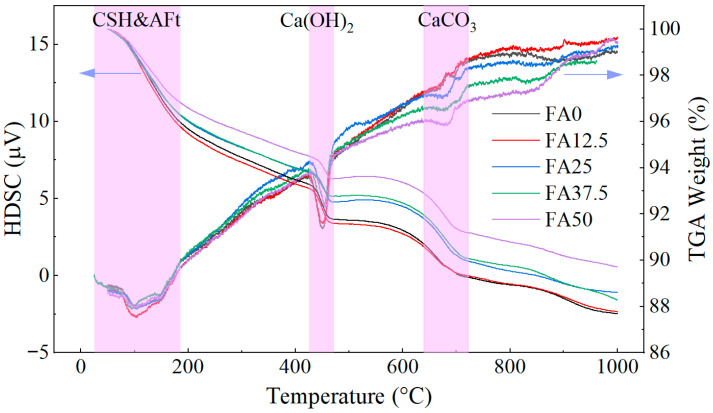
TG and HDSC curves of ECC.

To eliminate the influence of CaCO_3_ originally present in the cement on the calculated CH content, TGA was also performed on the cement. The TG and DSC curves of cement and ECC are presented in Figure 12 [36] and Figure 13, respectively. The contents of CH, CaCO_3_, and chemically bound water in C-S-H and Aft C-S-H, were calculated according to Equations (19)–(21).

(19) $$w_{H_{2}O(\mathrm{CSH}+\mathrm{AFt})}=w_{50\;{}^{\circ}C\sim 190\;{}^{\circ}C}$$

(20) $$w_{\mathrm{CH}}=\frac{74}{18}w_{420\;{}^{\circ}C\sim 470\;{}^{\circ}C}+\frac{74}{44}w_{\mathrm{CC}}$$

(21) $$w_{\mathrm{CC}}=\frac{100}{44}w_{640\;{}^{\circ}C\sim 720\;{}^{\circ}C}-w_{\mathrm{CC}(\mathrm{cement})}$$

where $w_{H_{2}O(\mathrm{CSH}+\mathrm{AFt})}$—mass fraction of chemically bound water in C-S-H and Aft in the sample (%);

$w_{a\;{}^{\circ}C\sim b\;{}^{\circ}C}$—mass loss fraction of sample from a °C to b °C (%);
$w_{\mathrm{CH}}$—mass fraction of CH in the sample (%);
  $w_{\mathrm{CC}}$—mass fraction of CaCO_3_ in the sample (%),
$w_{\mathrm{CC}(\mathrm{cement})}$—mass fraction of CaCO_3_ in cement (%).

**Figure 14:**
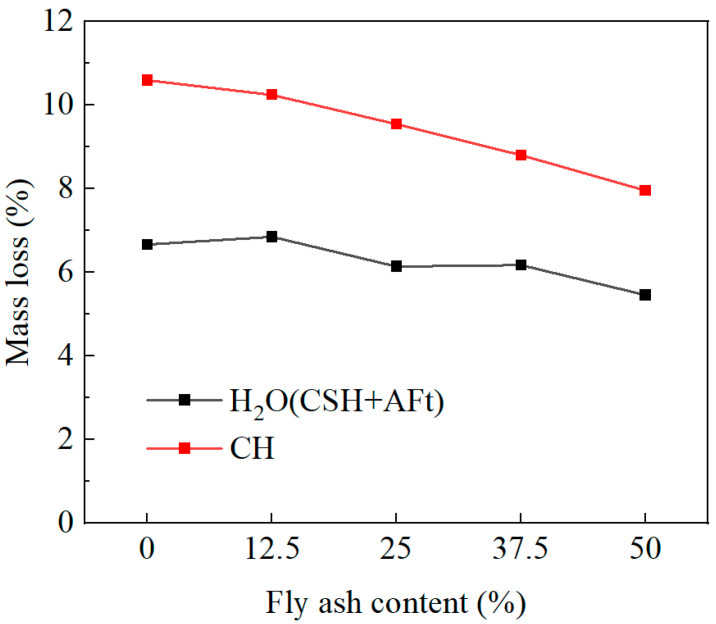
Mass loss of CH and chemically bound water associated with C-S-H and AFt.

Figure 14 presents the contents of CH and chemically bound water associated with C-S-H and AFt. The content of chemically bound water initially increases and then decreases with fly ash content, reaching a maximum at 12.5%. This trend is consistent with the variation in *τ*_0_. At low fly ash contents, the reduction in clinker decreases primary hydration products, but this is compensated by secondary pozzolanic reactions consuming CH and generating additional C-S-H. At higher fly ash contents, however, the reduced cement content limits hydration, and the pozzolanic reaction becomes insufficient to compensate, leading to a net decrease in hydration products. Meanwhile, CH content decreases continuously due to both reduced cement hydration and its consumption in pozzolanic reactions.

### 4.4. Comprehensive Evaluation of Mechanical Performance

**Figure 15:**
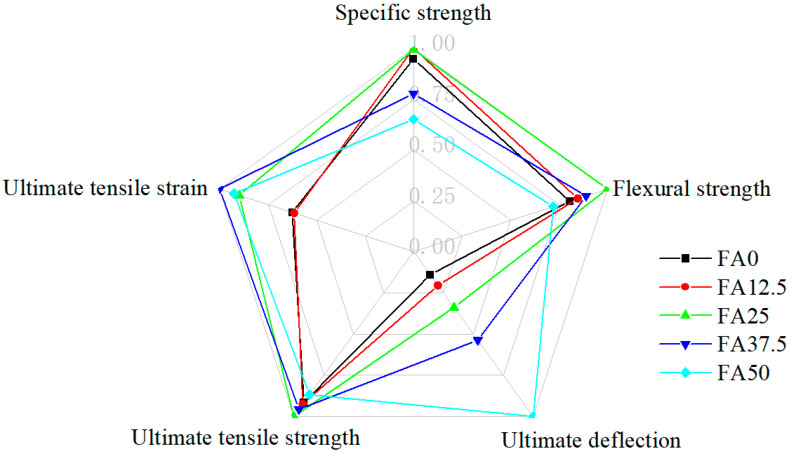
General comparison of mechanical properties of ECC with varying fly ash content.

To compare overall mechanical performance, key parameters were normalized by their respective maximum values and visualized using radar charts (Figure 15). The enclosed area represents the combined performance of the strength and ductility.

After interfacial optimization, all mixtures show improved performance. Among them, FA25, FA37.5, and FA50 exhibit the most favorable profiles. FA25 and FA37.5 achieve an optimal balance between strength and deformability, whereas FA50 provides the highest ductility at the expense of strength.

A quantitative comparison (Table 10) further highlights the effectiveness of the proposed strategy. Taking FA37.5 as a representative case, the optimized mixture achieved a 3.1% reduction in density and a 17.9% decrease in specific compressive strength relative to the unoptimized counterpart FA0. In contrast, flexural strength, ultimate deflection, ultimate tensile strength and tensile strain increased by 10.5%, 286.8%, 4.6% and 63.2%, respectively. Compared with the benchmark ECC-M45, FA37.5 achieved a 32% higher specific strength while using 40% less fiber, maintaining comparable tensile ductility and only a 12% reduction in ultimate tensile strength. Compared with lightweight PVA-ECC fabricated from similar materials, FA37.5 achieves higher specific compressive strength and comparable tensile strength and ductility, despite a substantially reduced fiber content.

## 5. Conclusions

To achieve strength–ductility balance and enhance the toughness of ECCs, this study establishes a micromechanics-based interfacial regulation framework centered on fiber–matrix frictional behavior. The main conclusions are summarized as follows:

(1) The fiber–matrix interfacial frictional stress *τ*_0_ is identified as a governing bifurcation parameter controlling the transition between fiber pull-out and fracture, which in turn controls the tensile toughness of ECC. A critical value, (*τ*_0_)_crit_, is defined as the bifurcation point at which maximum energy dissipation is achieved, providing a quantitative criterion for toughness enhancement in ECCs.
(2) A micromechanical model linking *τ*_0_ to the fiber bridging stress–crack opening displacement (*σ*-*δ*) relationship is established, and a simplified back-calculation approach is proposed to determine *τ*_0_ directly from single-crack tensile tests. This method enables reliable interfacial characterization without reliance on single-fiber pull-out experiments.
(3) The evolution of *τ*_0_ is governed by the content of hydration products, particularly C-S-H and AFt. The combined effects of cement hydration and pozzolanic reaction lead to a non-monotonic variation in τ_0_ with fly ash content. Maximum toughness is achieved when *τ*_0_ approaches to (*τ*_0_)_crit_, experimentally validating the proposed micromechanical theory.
(4) Significant mechanical enhancement was achieved at reduced fiber dosage. By regulating *τ*_0_ through fly ash incorporation, the ECC with 37.5% fly ash achieves the optimal strength–ductility. Despite a reduced fiber content (1.2 vol%), the optimized ECC attains comparable tensile ductility and competitive strength relative to conventional ECCs with higher fiber dosage, demonstrating substantially improved material efficiency.

Overall, this study demonstrates that interfacial friction can be treated as a quantifiable and designable parameter for controlling fiber failure modes and optimizing ECC performance. The proposed framework provides a mechanistically grounded and experimentally validated pathway for developing high-performance and low-fiber ECC materials.

## Figures and Tables

**Table 1:** Mix proportion of ECC-M45 (kg/m^3^) [3].

Mix	Cement	Fly Ash	Water	PVA Fiber	Silica Sand	WRA
ECC-M45	570	684	331	26	455	4.9

**Table 2:** Mechanical properties of ECC-M45 [3].

Mix	Density	Compressive Strength (MPa)		Ultimate Strain	Ultimate Stress
	(kg/m^3^)	7 d	28 d	(%)	(MPa)
ECC-M45	2071	38.1	50.2	3.16 ± 0.68	4.45 ± 0.11

**Table 3:** Oxide composition of cement, fly ash and FAC (wt.%).

Powder	SiO_2_	Al_2_O_3_	Fe_2_O_3_	CaO	MgO	*f*-CaO	SO_3_
Cement	50.1	23.9	7.65	11.9	1.96	1.01	0.98
Fly ash	56.7	24.6	6.55	4.87	N.A.	N.A.	N.A.
FAC	50–60	35–38	1.0–4.0	1.5–4.0	0.8–1.5	N.A.	N.A.

**Table 4:** Mix proportions of ECC (as mass ratio of binder).

Mix ID	Water	Binder			FAC	Fiber (Vol.%)	WRA	Density (kg/m^3^)	Compressive Strength (MPa)
		Cement	Fly Ash	Silica Fume					
FA0	0.25	0.95	0	0.05	0.3	1.2	0.03	1556	60.7
FA12.5	0.25	0.825	0.125	0.05	0.3	1.2	0.025	1548	63.7
FA25	0.25	0.7	0.25	0.05	0.3	1.2	0.025	1525	62.3
FA37.5	0.25	0.575	0.375	0.05	0.3	1.2	0.02	1507	48.2
FA50	0.25	0.45	0.5	0.05	0.3	1.2	0.02	1501	40.2

**Table 5:** Parameters from matrix fracture toughness test.

Fly Ash Content (%)	Mass (kg)	Peak Load (kN)	*K_m_* (MPa·m^0.5^)
0	0.403	0.903	0.572
12.5	0.402	0.901	0.570
25	0.373	0.816	0.517
37.5	0.373	0.794	0.503
50	0.372	0.717	0.456

**Table 6:** Fiber bridging parameters obtained via single-crack tensile tests.

Fly Ash Content (%)	*σ*_0_ (MPa)	*δ*_0_ (mm)	$\int_{0}^{\delta_{0}}\sigma (\delta )d\delta$ (J/m^2^)
0	4.14	0.202	0.689
12.5	4.40	0.248	0.902
25	4.58	0.298	1.111
37.5	4.50	0.336	1.212
50	4.41	0.319	0.737

**Table 7:** Mechanical parameters of ECC under uniaxial tension [36].

Fly Ash Content (%)	First Crack Strength	Ultimate Strain	Ultimate Stress
	(MPa)	(%)	(MPa)
0	2.85 ± 0.18	1.92 ± 0.17	3.73 ± 0.12
12.5	2.84 ± 0.15	1.89 ± 0.18	3.77 ± 0.15
25	2.55 ± 0.13	2.76 ± 0.61	4.08 ± 0.19
37.5	2.51 ± 0.10	3.07 ± 0.42	3.90 ± 0.15
50	2.12 ± 0.05	2.84 ± 0.70	3.54 ± 0.22

**Table 8:** σ_fc_,σ_0_ and PSH_s_.

Fly Ash Content (%)	*σ_fc_* (MPa)	*σ*_0_ (MPa)	*PSH_s_*
0	2.85	4.14	1.45
12.5	2.84	4.40	1.55
25	2.72	4.58	1.80
37.5	2.56	4.50	1.79
50	2.12	4.41	2.08

**Table 9:** *J_tip_*, *J_b_*′ and *PSH_e_*.

Fly Ash Content (%)	*J_tip_* (J/m)	*J_b_*′ (J/m)	*PSH_e_*
0	21.03	148.8	7.07
12.5	18.58	196.3	10.57
25	17.63	233.7	13.26
37.5	16.24	220.0	13.55
50	13.47	266.8	19.81

**Table 10:** General comparison between mechanical properties before and after optimization and lightweight PVA-ECC in relative studies.

Mix	Fiber Content (Vol.%)	Density (kg/m^3^)	Specific Strength (kPa·m^3^/kg)	Flexural Strength (MPa)	Ultimate Deflection (mm)	Ultimate Tensile Strength (MPa)	Ultimate Tensile Strain (%)
Control	1.2	1556	39.0	9.5	0.53	3.73	1.9
FA37.5	1.2	1507	32.0	10.5	2.05	3.90	3.1
ECC-M45	2.0	2071	24.2	—	—	4.45	3.2
Wang [37]	1.75 (of cement)	1260~1610	6.2~25.5	7.4~15.4	—	—	—
Zhu [38]	1.75	1300~1400	10.0~23.8	—	—	3.00	2.0~4.0
AbdelAleem [39]	2.0	1740~1835	18.7~23.5	6.3~9.7	—	—	—
Ma [40]	2.0	1875	8.8			2.73	1.0
Huang [41]	2.0	1698	26.1	—	—	5.60	3.6
		1649	15.2	—	—	4.80	4.3

## Data Availability

The original contributions presented in this study are included in the article. Further inquiries can be directed to the corresponding author.
